# Supplementary material for: Harnessing the power of comparative genomics to support the distinction of sister species within Phyllosticta and development of highly specific detection of Phyllosticta citricarpa causing citrus black spot by real-time PCR
Source: PeerJ. 2023 Oct 23;11:e16354. doi: 10.7717/peerj.16354 (PMC10601906; doi:10.7717/peerj.16354)
Supplement: Supplemental Information 3 — Accessions in red indicate the sequences that were submitted to Genbank. Accessions in blue indicate that the isolate has the same sequence that the one submitted (indicated in red) to Genbank. Accessions in green indicate the sequences that were unique to the species, and submitted to Ganbank. Accessions in black are from other studies. [file peerj-11-16354-s003.docx]

**Supplemental information 3**

**Supplementary table 1. Microsatellite multilocus profile for the ten single sequence repeat (SSR) markers developed by [1] and [2]**

| **Identity** | **Isolate** | **PC6^1^** | **PC37^1^** | **PC322^1^** | **Pc7^1^** | **Pc20^1^** | **Pc440^2^** | **Pc117^2^** | **Pc1007^2^** | **Pc19^1^** | **Pc12^1^** |
| --- | --- | --- | --- | --- | --- | --- | --- | --- | --- | --- | --- |
| ***P. citricarpa*** | **LSVM359** | 126 | 147 | 152 | 171 | 204 | 266 | 178 | 155 | 160 | 182 |
| ***P. citricarpa*** | **LSVM1101** | 129 | 147 | 152 | 183 | 204 | 266 | 178 | 155 | 160 | 182 |
| ***P. citricarpa*** | **LSVM1123** | 118 | 147 | 152 | 183 | 204 | 266 | 178 | 155 | 160 | 182 |
| ***P. citricarpa*** | **LSVM1499** | 126 | 147 | 152 | 183 | 204 | 266 | 178 | 155 | 160 | 182 |
| ***P. citricarpa**** | **CBS141350** | 118 | 147 | 152 | 171 | 204 | Missing | 178 | 158 | 157 | 182 |
| ***P. paracitricarpa**** | **CBS141357** | 135 | 144 | 160 | 177 | 200 | 266 | 178 | 160 | 160 | 173 |
| ***P. paracitricarpa**** | **CBS141358** | 135 | 144 | 160 | 177 | 200 | 269 | 178 | 158 | 160 | 173 |
| ***P. paracitricarpa**** | **ZJUCC20093** | 135 | 144 | 160 | 177 | 200 | 269 | 178 | 158 | 160 | 173 |
| ***P. paracitricarpa*** | **GIHF303** | 141 | 144 | 160 | 177 | 200 | 269 | 178 | 158 | 157 | 173 |
| ***P. paracitricarpa*** | **LSVM1238** | 135 | 144 | 160 | 177 | 194 | 266 | 149 | 158 | 157 | 190 |

[1] N-Y Wang et al., Phytopathology® 106 (11), 1300 (2016).

[2] E Carstens et al., Phytopathology 107 (6), 758 (2017).

* According to V Guarnaccia et al., Studies in Mycology 87, 161 (2017).

**Supplementary figure 1. Minimum spanning network (MSN)**. **The minimum spanning network was constructed based on pairwise allele shared distance. Computed distances are indicated alongside the edges of the MSN.**


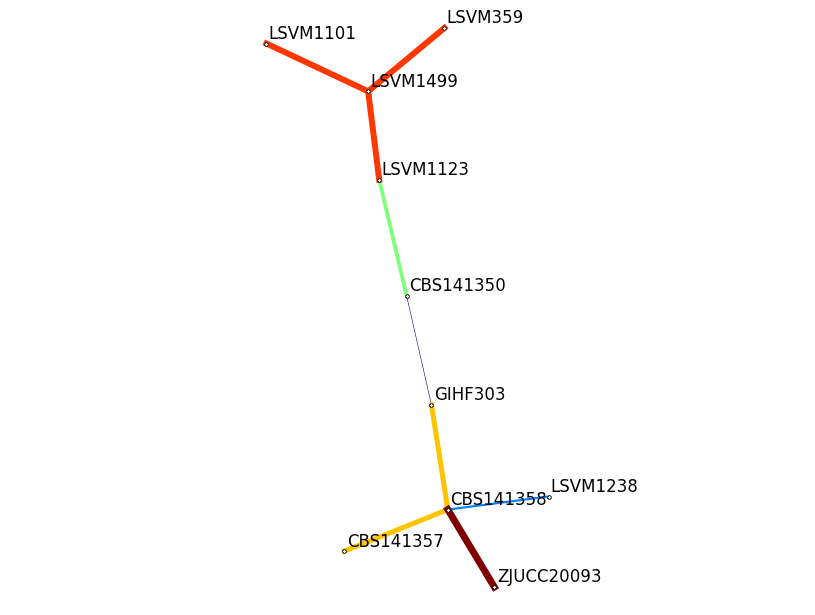


0.67

0.20

0.20

0.50

0.0

0.22

0.10

0.10

0.10

*Phyllosticta citricarpa*

*Phyllosticta paracitricarpa*
