# Supplementary material for: Harnessing the power of comparative genomics to support the distinction of sister species within Phyllosticta and development of highly specific detection of Phyllosticta citricarpa causing citrus black spot by real-time PCR
Source: PeerJ. 2023 Oct 23;11:e16354. doi: 10.7717/peerj.16354 (PMC10601906; doi:10.7717/peerj.16354)
Supplement: Supplemental Information 4 [file peerj-11-16354-s004.docx]

**Supplemental information 4: assessment of repeatability and reproducibility of the qCBS real-time protocol**

|  |  | **Repeatability** | | | **Reproducibility** | | |
| --- | --- | --- | --- | --- | --- | --- | --- |
| **DNA template** | **Concentration** | **Mean Ct** | **SD** | **CV (%)** | **Mean Ct** | **SD** | **CV (%)** |
| G23 target plasmid DNA | 10xLOD^a^ | 33.6 | 0,4 | 1,19 | 33.7 | 1.17 | 3.49 |
| G23 target plasmid DNA | 100xLOD^a^ | 29.86 | 0,41 | 1,37 | 29.86 | 1.32 | 4.41 |
| G23 target plasmid DNA | 1000xLOD^a^ | 26.09 | 0,28 | 1,07 | n.d. | n.d. | n.d. |
| *P. citricarpa* LSVM 1501 | 0,1 ng/µL + 1 ng/µL orange | 27.83 | 0,16 | 0,57 | 28.77 | 1.03 | 3.59 |
| *P. citricarpa* LSVM 1501 | 0,1 ng/µL + 1 ng/µL lemon | 28.33 | 0,15 | 0,53 | 28.57 | 1.17 | 4.09 |
| *P. citriasiana* LSVM 1146 | 1 ng/µL | >45 | - | - | >45 | - | - |
| *P. paracitricarpa* LSVM 1238 | 1 ng/µL | >45 | - | - | >45 | - | - |
| *P. paracitricarpa* ZJUCC200937 | 1 ng/µL | >45 | - | - | >45 | - | - |

^a^ The concentration used as the limit of detection (LOD) corresponds to 31.6 pc/µL^-1^
