# Supplementary material for: Harnessing the power of comparative genomics to support the distinction of sister species within Phyllosticta and development of highly specific detection of Phyllosticta citricarpa causing citrus black spot by real-time PCR
Source: PeerJ. 2023 Oct 23;11:e16354. doi: 10.7717/peerj.16354 (PMC10601906; doi:10.7717/peerj.16354)
Supplement: Supplemental Information 5 — The table reports mean Ct values generated when subtle alterations of PCR reaction volume or temperature were implemented, with the same set of templates. Parameters in bold characters correspond to the optimized conditions for the assay. [file peerj-11-16354-s005.docx]

**Supplemental information 5: Assessment of robustness of the qCBS real-time protocol. The table reports mean Ct values generated when subtle alterations of PCR reaction volume or temperature were implemented, with the same set of templates. Parameters in bold characters correspond to the optimized conditions for the assay.**

| **Hybridization temperature** | **68°C** | 68°C | 68°C | 66°C | 70°C |
| --- | --- | --- | --- | --- | --- |
| **Reaction volume** | **20 µL** | 18 µL | 22 µL | 20 µL | 20 µL |
| **DNA template (Concentration)** |  |  |  |  |  |
| G23 target plasmid DNA(10xLOD^a^) | 33.60±0.40 | 34.45±0.36 | 34.82±0.64 | 30.31±0.48 | >45 |
| G23 target plasmid DNA (100xLOD^a^) | 29.86±0.41 | 30.79±0.26 | 31.34±0.20 | 26.71±0.23 | >45 |
| *P. citricarpa* LSVM 1501 (0.1 ng/µL + 1 ng/µL orange) | 27.83±0.16 | 28.62±0.20 | 29.05±0.31 | 25.55±0.12 | 40.83±0.91 |
| *P. citricarpa* LSVM 1501 (0.1 ng/µL + 1 ng/µL lemon) | 28.33±0.15 | 29,37±0.20 | 29.75±0.16 | 25.63±0.17 | >45*** |
| *P. citriasiana* LSVM 1146 (1 ng/µL) | >45 | >45* | >45** | 35.28±0.52 | >45 |
| *P. paracitricarpa* LSVM 1238 (1 ng/µL) | >45 | >45 | >45 | >45 | >45 |
| *P. paracitricarpa* ZJUCC200937 (1 ng/µL) | >45 | >45 | >45 | >45 | >45 |

^a^ The concentration used as the limit of detection (LOD) corresponds to 31.6 pc/µL^-1^

* Four out of ten replicates gave Ct values between 41.78 and 43.50

** One out of ten replicates gave a Ct value of 43.31

*** Four replicates out of the 10 gave Ct values between 43.25 and 44.82
