# Supplementary material for: Harnessing the power of comparative genomics to support the distinction of sister species within Phyllosticta and development of highly specific detection of Phyllosticta citricarpa causing citrus black spot by real-time PCR
Source: PeerJ. 2023 Oct 23;11:e16354. doi: 10.7717/peerj.16354 (PMC10601906; doi:10.7717/peerj.16354)
Supplement: Supplemental Information 6 — Different qPCR equipment and commercial qPCR master mixes were used with the same set of templates. [file peerj-11-16354-s006.docx]

**Supplemental information 6: Assessment of transferability of the qCBS real-time protocol. Different qPCR equipment and commercial qPCR master mixes were used with the same set of templates.**

|  |  | **No ROX core kit, Eurogentec**  **+ Rotorgene Q** | | | | **qPCR No ROX master mix, Eurogentec + Rotorgene Q** | | | | **QuantaBio perfeCTa qPCR ToughMix + Rotorgene Q** | | | | **No ROX core kit, Eurogentec**  **+ Roche LifeCycler480** | | | | **Takyon No ROX Probe Core Kit, Eurogentec + Rotorgene** | | | | **TaKaRa Premix Ex Taq Probe qPCR + Rotorgene Q (GIHF lab)** | | |
| --- | --- | --- | --- | --- | --- | --- | --- | --- | --- | --- | --- | --- | --- | --- | --- | --- | --- | --- | --- | --- | --- | --- | --- | --- |
| **DNA template** | **Concentration** | Ct | SD | CV | Ct | | SD | CV | Ct | | SD | CV | Ct | | SD | CV | Ct | | SD | CV | Ct | | SD | CV |
| G23 target plasmid DNA | 10xLOD^a^ | 33,60 | 0,40 | 1,19 | >45 | | n.a. | n.a. | 39,66 | | 0,39 | 0,98 | 33,2 | | 0,27 | 0,81 | 30.97 | | 0.35 | 1.13 | >45 | | n.a. | n.a. |
| G23 target plasmid DNA | 100xLOD^a^ | 29,86 | 0,41 | 1,37 | >45 | | n.a. | n.a. | 35,49 | | 0,29 | 0,82 | 30,01 | | 0,11 | 0,37 | 27.65 | | 0.15 | 0.54 | >45 | | n.a. | n.a. |
| *P. citricarpa* LSVM 1501 | 0,1 ng/µL + 1 ng/µL orange | 27,83 | 0,16 | 0,57 | >45 | | n.a. | n.a. | 32,1 | | 0,33 | 1,03 | 29,74 | | 0,07 | 0,24 | 25.14 | | 0.23 | 0.91 | >45 | | n.a. | n.a. |
| *P. citricarpa* LSVM 1501 | 0,1 ng/µL + 1 ng/µL lemon | 28,33 | 0,15 | 0,53 | >45 | | n.a. | n.a. | 33,31 | | 0,33 | 0,99 | 29,77 | | 0,12 | 0,4 | 25.26 | | 0.21 | 0.83 | >45 | | n.a. | n.a. |
| *P. citriasiana* LSVM 1146 | 1 ng/µL | >45 | n.a. | n.a. | >45 | | n.a. | n.a. | >45 | | n.a. | n.a. | >45 | | n.a. | n.a. | 33.31 | | 0.27. | 0.08 | >45 | | n.a. | n.a. |
| *P. paracitricarpa* LSVM 1238 | 1 ng/µL | >45 | n.a. | n.a. | >45 | | n.a. | n.a. | >45 | | n.a. | n.a. | >45 | | n.a. | n.a. | >45 | | n.a. | n.a. | >45 | | n.a. | n.a. |
| *P. paracitricarpa* ZJUCC200937 | 1 ng/µL | >45 | n.a. | n.a. | >45 | | n.a. | n.a. | >45 | | n.a. | n.a. | >45 | | n.a. | n.a. | >45 | | n.a. | n.a. | >45 | | n.a. | n.a. |

^a^ The concentration used as the limit of detection (LOD) corresponds to 31.6 pc/µL^-1^
