# Supplementary material for: Harnessing the power of comparative genomics to support the distinction of sister species within Phyllosticta and development of highly specific detection of Phyllosticta citricarpa causing citrus black spot by real-time PCR
Source: PeerJ. 2023 Oct 23;11:e16354. doi: 10.7717/peerj.16354 (PMC10601906; doi:10.7717/peerj.16354)
Supplement: Supplemental Information 7 — Read alignments of the strains belonging to the P. citricarpa and P. paracitricarpa populations were checked using the Integrative Genomics Viewer (IGV). [file peerj-11-16354-s007.pdf]

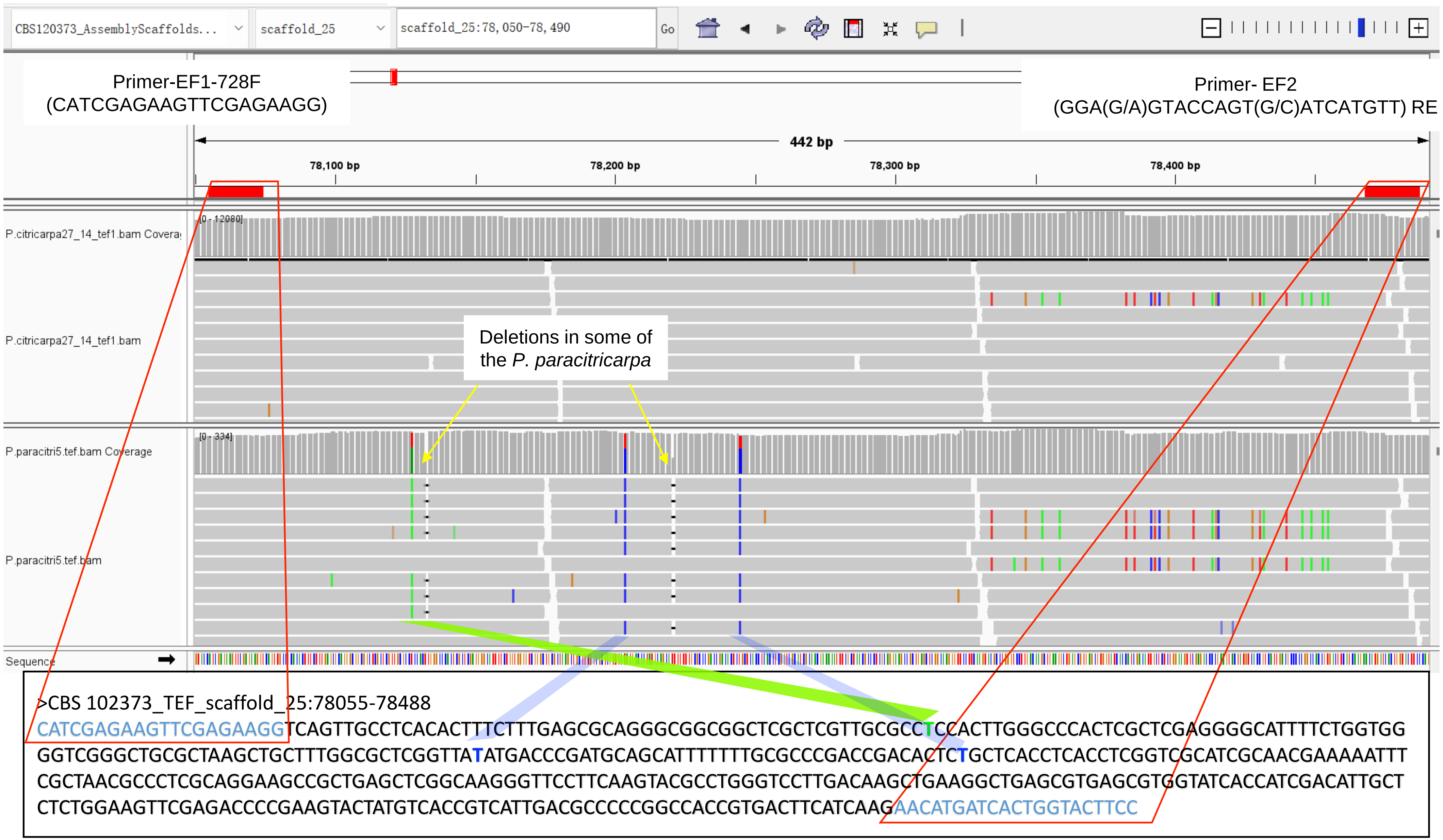

Supplemental information 7: Genomic variations present in the *P. paracitricarpa* isolates in the *tef1* locus. Read alignments of the strains belonging to the *P. citricarpa* and *P. paracitricarpa* populations were checked using the Integrative Genomics Viewer (IGV).
